# Status and trends in glioblastoma lipid metabolism research: a bibliometric analysis

**DOI:** 10.1007/s12672-026-05298-1

**Published:** 2026-05-30

**Authors:** Wenliang Wang, Ye Yuan, Ruohao Sun, Mavie Katalin Surau, Phillip Alexander Becker, Fu Xu, Mohammad Shah Nayaz Burkutally, Yuhang Chen, Long Chen

**Affiliations:** 1 Department of Neurosurgery, Union Hospital, Tongji Medical College, Huazhong University of Science and Technology, Wuhan, 430022 China; 2 Department of Neurosurgery, Beijing Tiantan Hospital, Capital Medical University, Beijing, 100070 China; 3 China National Clinical Research Center for Neurological Diseases, Beijing, 100070 China; 4 Clinic for Neurology, University Hospital Essen, University of Duisburg-Essen, Hufelandstraße 55, D-45147 Essen, Germany; 5 Department of Pediatrics, University Hospital Essen, University of Duisburg- Essen, Hufelandstraße 55, D-45147 Essen, Germany; 6 Department of Neurosurgery, The first Affiliate Hospital of Henan University of Science and Technology, Luoyang, 471000 China

**Keywords:** Glioblastoma, Lipid Metabolism, Bibliometrics Analysis, Metabolic Reprogramming

## Abstract

Glioblastoma multiforme (GBM), which is the most lethal brain tumor, exhibits high aggressiveness, recurrence, and poor treatment response. The progression of GBM and resistance to therapy is attenuated by metabolic reprogramming and lipid metabolism. In this study, we conducted a bibliometric analysis of GBM lipid metabolism studies starting from the mid-1990s based on 798 publications from Web of Science Core Collection. In particular, publication trends, geographic and institutional distribution, authorship networks, keyword co-occurrence, and citation bursts, using CiteSpace, VOSviewer, and R-bibliometrix, were examined. Studies published in the US and China increased substantially after 2010. *De novo* lipogenesis, lipid droplet biology, and treatment targeting of lipid regulatory processes, such as fatty-acid oxidation, were the most frequently studied topics. Recent interest in lipid–immune interactions, ferroptosis, and lipomics suggests that further research is required. This bibliometric overview provides a solid foundation for future studies of GBM lipid metabolism and its clinical translation.

**Supplementary Information:**

The online version contains supplementary material available at 10.1007/s12672-026-05298-1.

## Introduction

Glioblastoma multiforme (GBM) is the most commonly occurring primary malignant brain tumor, and accounts for 13.7% of all primary brain and other CNS tumors and 52.2% of malignant tumors (all ages, U.S.) based on the 2024 CBTRUS reports [1, 2]. It is characterized by microvascular proliferation and/or necrosis. WHO CNS5 defines it as “glioblastoma, IDH-wildtype, CNS WHO grade 4,” which is an IDH-wildtype and H3-wildtype diffuse astrocytic glioma, thus distinguishing it from other grade 4 astrocytoma-spectrum tumors [3–6]. Despite considerable progress in surgical resection, radiotherapy, and chemotherapy, particularly with temozolomide, the median overall survival for patients remains poor, ranging from 14 to 16 months [3, 7, 8]. This poor prognosis primarily results from diffuse infiltration, resistance to standard treatment, marked intratumoral heterogeneity, and extensive metabolic reprogramming [9, 10].

Metabolic reprogramming is a well-known feature of cancer. It supports tumors to meet the high bioenergetic and biosynthetic demands required by uncontrolled growth. The Warburg effect and glucose metabolism have long been a topic of interest, and lipid metabolism has recently become a relevant, but well-studied area of GBM [11]. Fernández-García et al. exploited the unique lipid metabolic dependencies of pediatric brain tumors for biomarker discovery. Targeted intervention holds greater promise compared with adapting conventional cancer therapies [12]. Lipids are essential for membrane production, energy storage, signaling, and cell fate decisions [13]. In the unique microenvironment of the central nervous system, GBM cells are highly adaptable and regulate lipid synthesis, uptake, oxidation, and storage to promote tumor growth [14].

Recent studies have examined metabolic pathways, such as de novo fatty-acid synthesis, fatty-acid oxidation (FAO), lipid droplets, cholesterol homeostasis, and lipase signaling [15–20]. These are regulated by transcriptional factors, such as sterol regulatory element-binding proteins, peroxisome proliferator-activated receptors, and hypoxia-inducible factors. Enzymes, such as fatty-acid synthase (FASN), acetyl-CoA carboxylase (ACC), diacylglycerol O-acyltransferase 1 (DGAT1), and sterol O-acyltransferase 1 (SOAT1), enable GBM to survive in nutrient-restricted hypoxic environments [11, 17, 21, 22]. These lipid modifications also enhance membrane biogenesis and energy storage.

Lipid metabolism is also important in immune resistance and tumor invasion [23]. Recent studies have also emphasized the connection between lipid metabolism and the immune microenvironment [24]. Lipid-rich tumor-associated macrophages, dysregulated arachidonic acid metabolism, and altered lipid antigen presentation have been observed [25, 26]. Moreover, lipid peroxidation and resistance to ferroptosis (a regulated iron-dependent cell death) are of interest in understanding treatment failure in GBM [14]. Therefore, lipids are both indicators of aggressive tumor activity and potential modulators of the immune response and therapy.

Although a mechanistic understanding of lipid metabolism in glioblastoma has progressed, a comprehensive picture of the research trends, collaborations, and future areas is not available. Bibliometric analysis provides a method to quantitatively analyze the development of a scientific research field over time. The analysis of publication trends, citation patterns, co-authorship patterns, and keyword evolution can detect influential authors, research hotspots, and knowledge gaps [27].

In this study, we present a bibliometric and qualitative description of GBM lipid metabolism research spanning nearly three decades. We applied data collected from the Web of Science Core Collection and used CiteSpace, VOSviewer, and R to quantify the intellectual structure and the dynamics of this research field. Our results may guide future studies by highlighting emerging ideas, translational challenges, and open challenges in glioblastoma lipid metabolism.

## Materials and methods

### Data source and search strategy

Bibliographic data were retrieved from the Web of Science Core Collection (WoSCC), which is a highly regarded and widely used database for bibliometric research because of its curated indexing and standardized metadata. For each study, we extracted bibliographic and analytical information, including the title, authors, author affiliations, publication year, journal, document type, abstract, keywords, cited references, total citation counts, and country/region information. The search was performed on July 31, 2025, and included publications from January 1, 1996, to July 31, 2025, to capture a complete longitudinal perspective of research in this field.

Lipid metabolism-related terms were selected to ensure comprehensive coverage of key lipid metabolic processes associated with glioblastoma biology, including lipid synthesis, FAO, lipid storage, and lipid signaling pathways, and encompassing both classical and emerging research themes. The query used canonical pathway/process terms to ensure broad coverage of lipid metabolism mechanisms in GBM, while avoiding narrow, intervention-specific phrasing that is inconsistently indexed among publications. The search strategy was formulated using topic terms (TS) to ensure the inclusion of the relevant literature at the intersection of glioblastoma and lipid metabolism. The final search query was as follows:

TS = ((glioblastoma* OR glioblastoma multiform OR glioblastoma multiforme OR grade IV glioma OR GBM) AND ((lipid metabolism) OR (fatty acid metabolism) OR (cholesterol metabolism) OR (lipid droplet*) OR phospholipid metabolism) OR (sphingolipid metabolism) OR (lipogenesis) OR (lipolysis) OR (fatty acid oxidation) OR (FAO) OR (acetyl-CoA)).

Only English-language publications were included. Eligible document types were limited to original research articles and reviews. Editorials, meeting abstracts, letters, book chapters, and other non-peer-reviewed formats were excluded.

### Inclusion and exclusion criteria

Studies were included if they were focused on lipid metabolism in glioblastoma, whether through a mechanistic, translational, preclinical, or clinical perspective. Articles that discussed tumor metabolism more broadly, without specifically focusing on lipid-related pathways, were excluded.

### Bibliometric tools and analytical framework

**Fig. 1:**
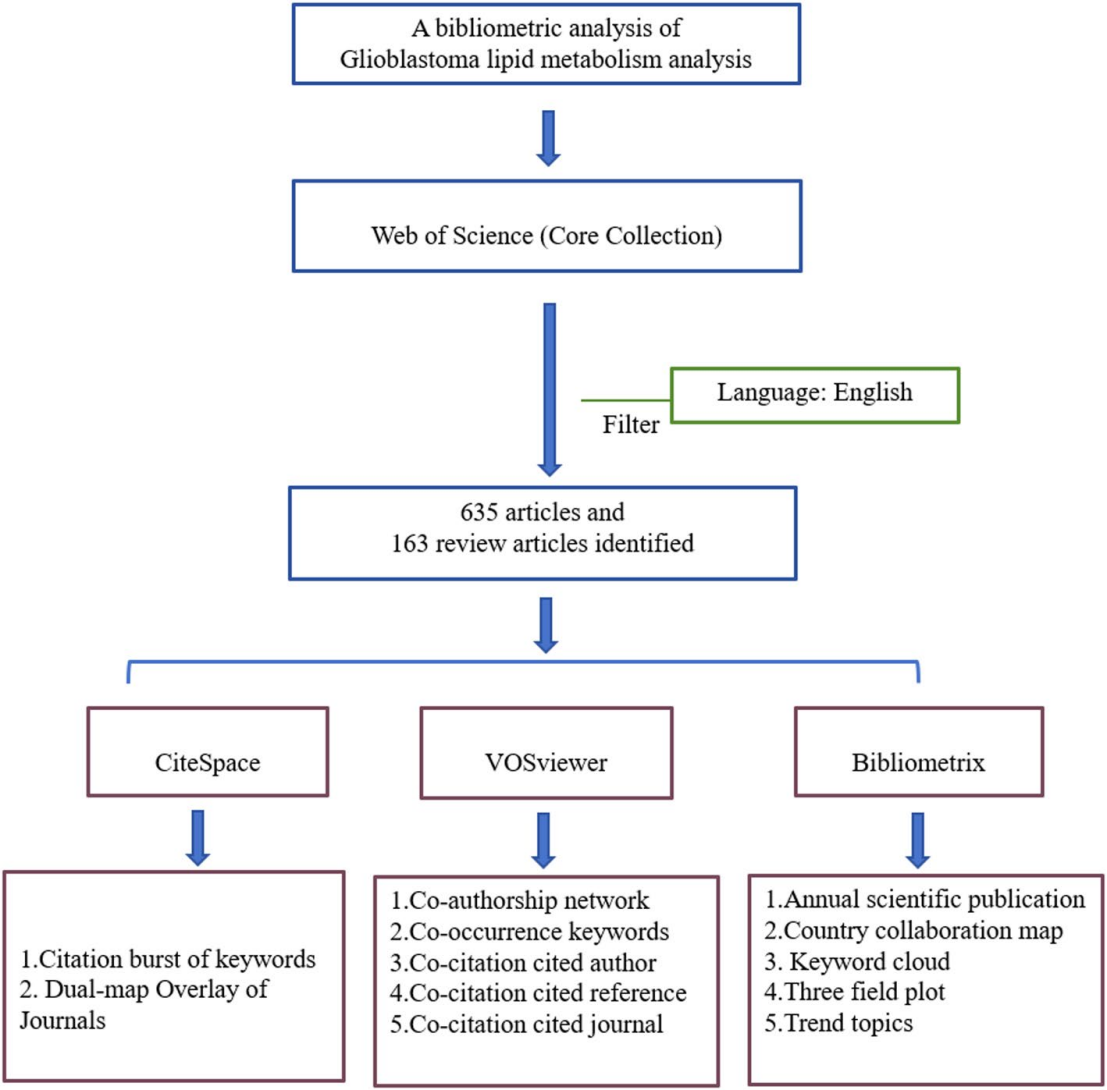
Workflow of the bibliometric analysis. A schematic diagram outlining the study design and methodology. The process included literature retrieval from the Web of Science Core Collection (WoSCC), data cleaning and selection, and subsequent analysis using CiteSpace, VOSviewer, and R-bibliometrix

Three validated software tools were used to conduct complementary layers of bibliometric analysis: CiteSpace (version 6.1.R6) was used for co-citation network analysis, citation burst detection, and cluster mapping to identify emerging research frontiers and thematic evolution [28]. VOSviewer (version 1.6.19, https://www.vosviewer.com) facilitated the construction of visual networks illustrating author collaboration, institutional productivity, country-level contributions, and keyword co-occurrence [29]. The Bibliometrix R-package (version 4.1.2, https://www.bibliometrix.org/), operating within the R statistical environment, was used for the descriptive analytics of annual publication trends, prolific journals, author productivity, and source impact [30]. Figures were generated using ggplot2 for data visualization. The workflow of the bibliometric analysis is shown in Fig. 1.

## Results

### Annual scientific output

**Fig. 2:**
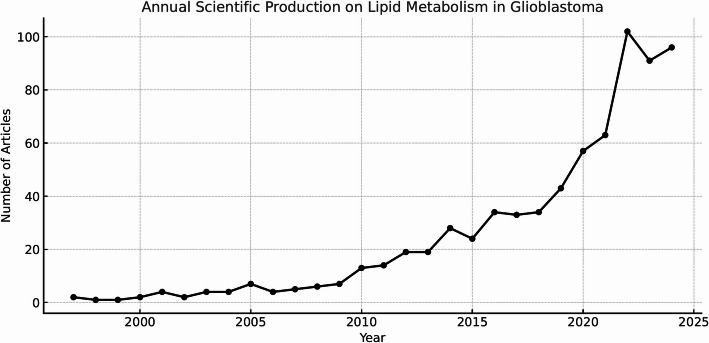
Annual publication trends on lipid metabolism in glioblastoma. The number of publications per year demonstrates a significant upward trajectory, particularly after 2010. The trend indicates the research attention on lipid-related mechanisms in GBM

Following manual screening and validation by two independent reviewers, 798 publications were retrieved, which consisted of 635 original research articles and 163 review articles. The full list of articles considered for inclusion is available in Supplementary File S1. The temporal trajectory of publications on glioblastoma lipid metabolism revealed an upward trend beginning in 1997. The initial period (1997–2009) was characterized by sporadic, low-volume output, which is indicative of nascent interest in lipid metabolic pathways in GBM. Around 2010, the annual number of publications began to steadily increase, paralleling the recognition of cancer metabolism as a therapeutic target. From 2017 onward, research activity markedly accelerated, reaching a peak in 2022 and 2023. This growth reflects a robust and expanding scientific community dedicated to uncovering the metabolic basis of GBM (Fig. 2).

### Country and institutional contributions

According to the contributing countries, the United States continues to provide important work in glioblastoma lipid metabolism. China has published a substantial amount in recent years, which has contributed to its leading position. Other contributing countries include Germany, the United Kingdom, and Italy.

**Fig. 3:**
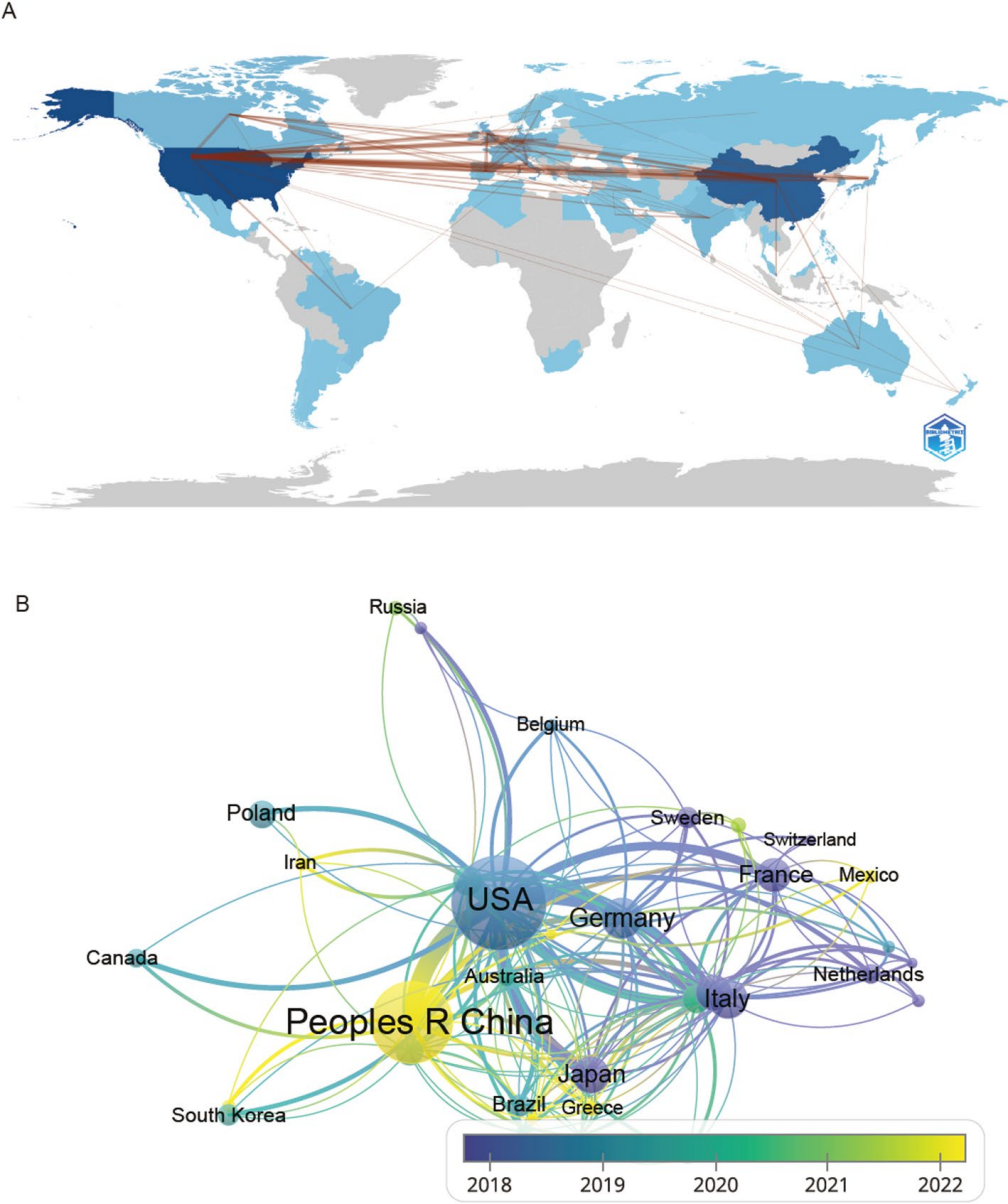
Global contributions and international collaboration network. **A** Geographical distribution of publications. The United States and China are leading contributors, followed by Germany, the United Kingdom, and Italy. Node size represents publication count; line thickness reflects collaboration strength. **B** Co-authorship network map showing inter-country collaboration in GBM lipid metabolism research. Strong collaborative ties are evident between the United States, European nations, and East Asia

As shown in Fig. 3A, the global collaboration map depicts a large co-authorship between the United States and other European countries because of strong transatlantic collaborations; however, Fig. 3B shows that although China published more frequently than Western countries in the Asian-Pacific region, the research collaborations are more concentrated in Asia-Pacific countries, and lack international relationships when compared to Western countries. Interestingly, the overlay suggests that publications from China and several Central/South American countries have a later average publication year, indicating that their contributions are more concentrated in recent years, consistent with a late growth phase in this subfield.

**Fig. 4:**
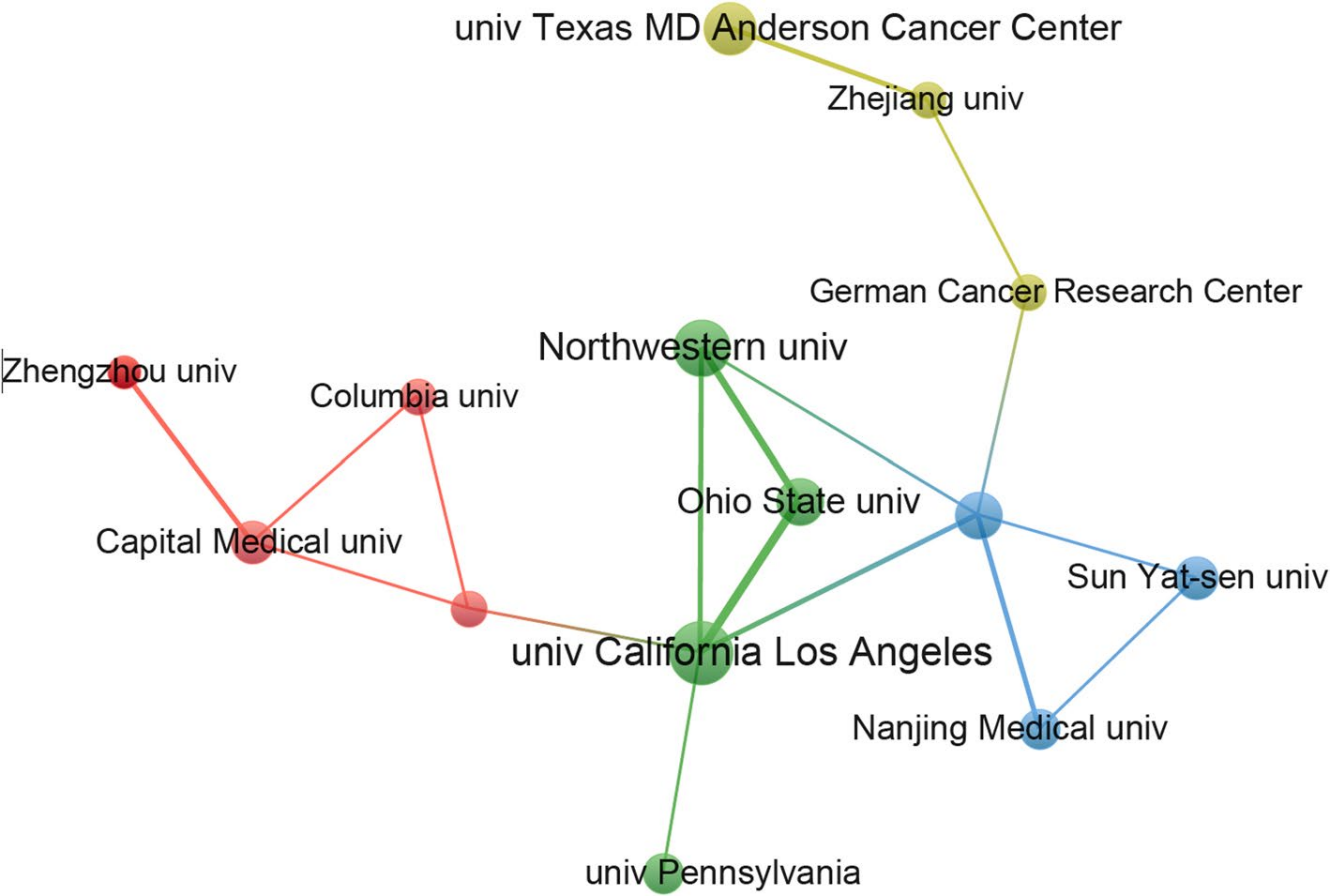
Institutional collaboration network. Network visualization of top contributing institutions. The Ohio State University, Fudan University, and Pécs Medical School form key hubs in the collaborative landscape

At the institutional level (Fig. 4), major academic research centers, such as the University of California, Los Angeles, Ohio State University, Northwestern University, and MD Anderson Cancer Center, have formed strong collaborations. In China, Capital Medical University, Zhejiang University, and Sun Yat-sen University form tightly-knit clusters, which suggests that domestic research collaborations play active roles. These patterns of institutional collaboration highlight the growing structural complexity and international research interest in glioblastoma lipid metabolism.

### Journal and citation analysis

Cancers (MDPI), Biomedicines, Cell Metabolism, Nature Reviews Cancer, and Cancer Research have emerged as prominent publications for studies on glioblastoma lipid metabolism. These journals differ in their thematic focus. For example, Cancers and Biomedicines frequently publish both clinical and translational research, whereas Cell Metabolism and Cancer Research tend to emphasize molecular and biochemical studies.

**Fig. 5:**
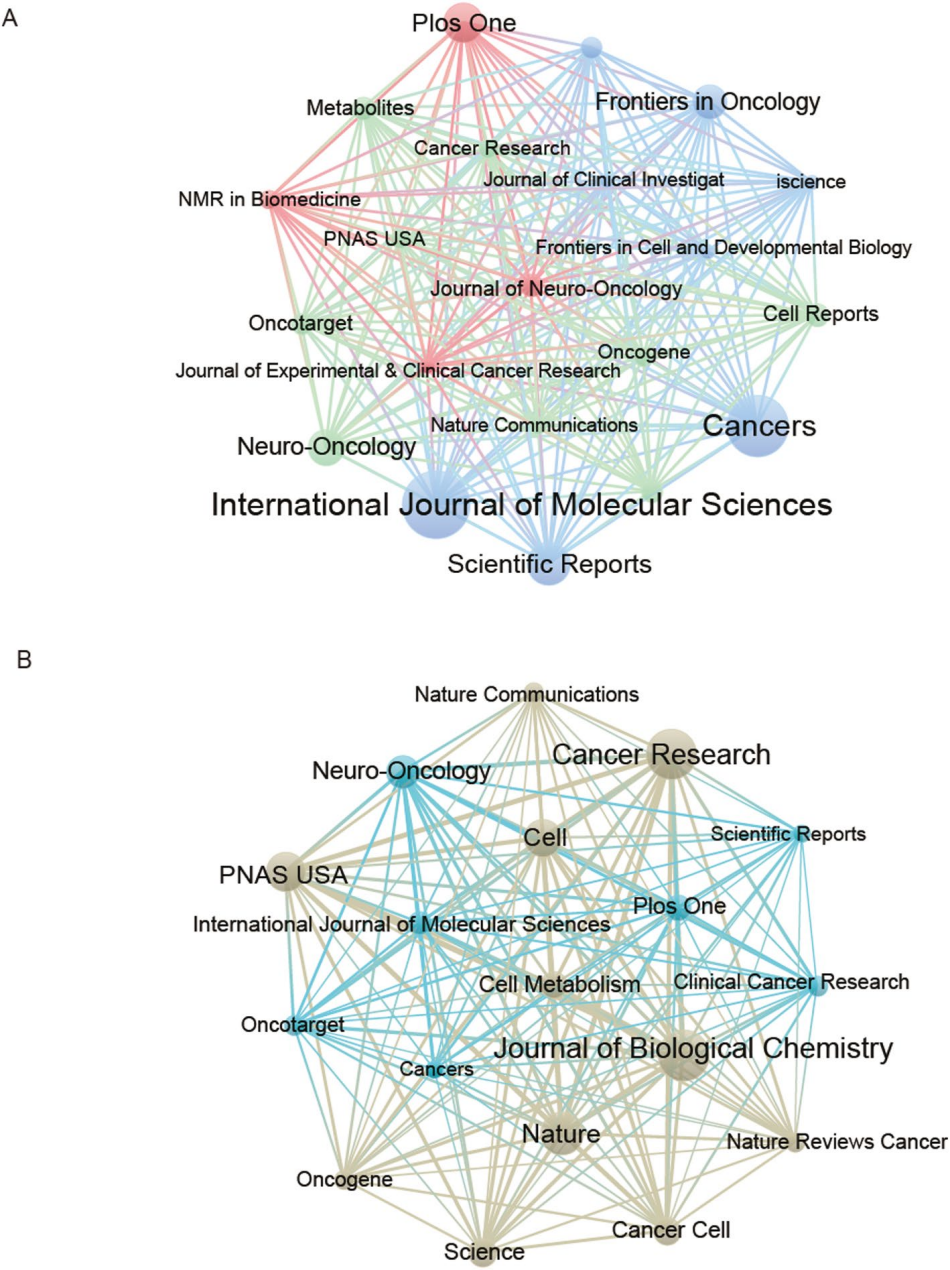
Journal analysis based on bibliographic coupling and co-citation. **A** Bibliographic coupling map generated by VOSviewer showing clusters of journals with similar citation behavior. Node size reflects the total number of citations, and node proximity represents similarity in citation patterns. **B** Co-citation network analysis identifying journals that are frequently cited together. Larger nodes indicate higher co-citation frequency

As shown by a journal coupling analysis (Fig. 5A), publication venues were grouped into clusters based on shared citation patterns, suggesting disciplinary convergence among oncology, metabolism, and molecular biology. The co-citation map (Fig. 5B) reveals the central role of high-impact journals, such as Nature, Cell, Journal of Biological Chemistry, and Cancer Cell, in shaping the intellectual landscape. These patterns reflect a multidisciplinary foundation and highlight the growing integration of metabolic research into neuro-oncology literature.

### Overlay visualization of disciplinary structure

**Fig. 6:**
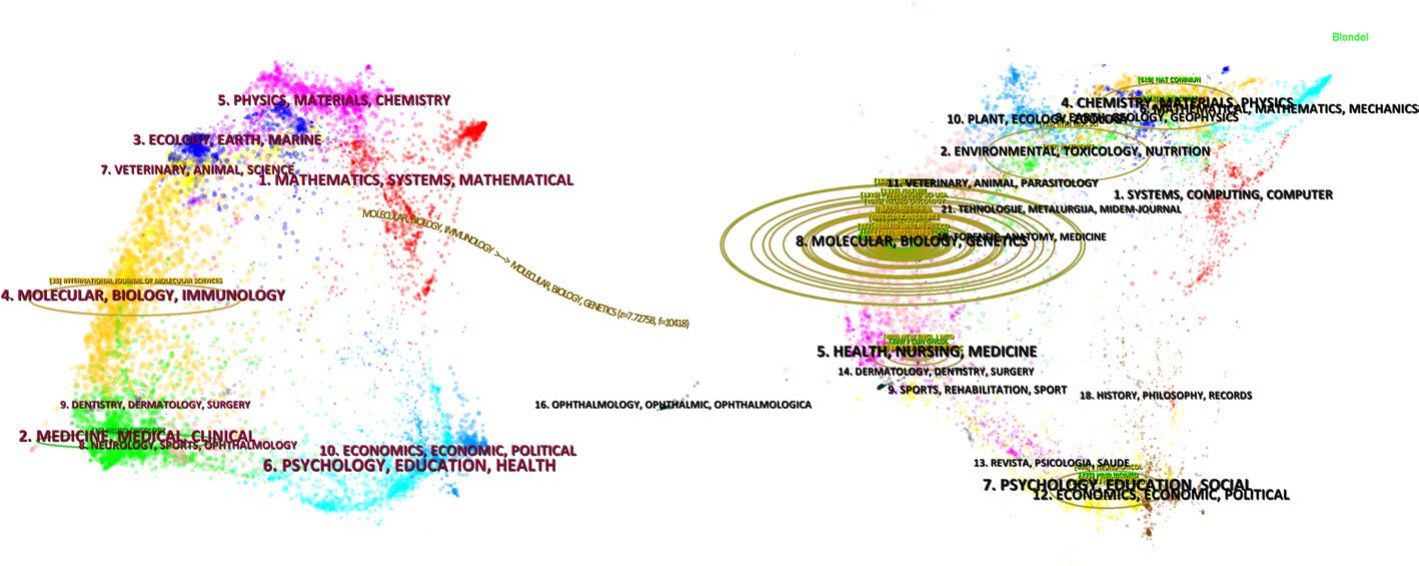
Dual-map overlay of citing and cited journals. A global citation map showing disciplinary cross-talk. Arrows point from citing journals (left) to cited journals (right), with major flows between oncology, molecular biology, and neurology fields

Figure 6 provides an overlay analysis showing the disciplinary and thematic structure of lipid metabolism studies in glioblastoma. The map integrates citation flows and topic evolution among the Web of Science subject categories. The field is anchored primarily in molecular biology, biochemistry, oncology, and immunology, as indicated by the dense clustering and strong linkages in these areas. These foundational disciplines serve as central hubs for cross-disciplinary integration.

The most prominent citation pathways were observed in molecular and cellular biology toward clinical medicine, suggesting a translational trajectory from mechanistic insights to applied therapeutic studies. Additional overlays extend into pharmacology, neuroscience, and bioengineering, which reflect the adoption of advanced tools, such as lipidomics, nanotechnology, and systems biology.

Emerging interdisciplinary linkages connect the core field to health sciences, environmental toxicology, and computational biology, thus revealing the expanding influence of glioblastoma lipid metabolism research into adjacent biomedical domains. This underscores the dynamic and integrative nature of the field, from its evolution as a molecular niche to a broad-based, translational research area.

### Author analysis

**Fig. 7:**
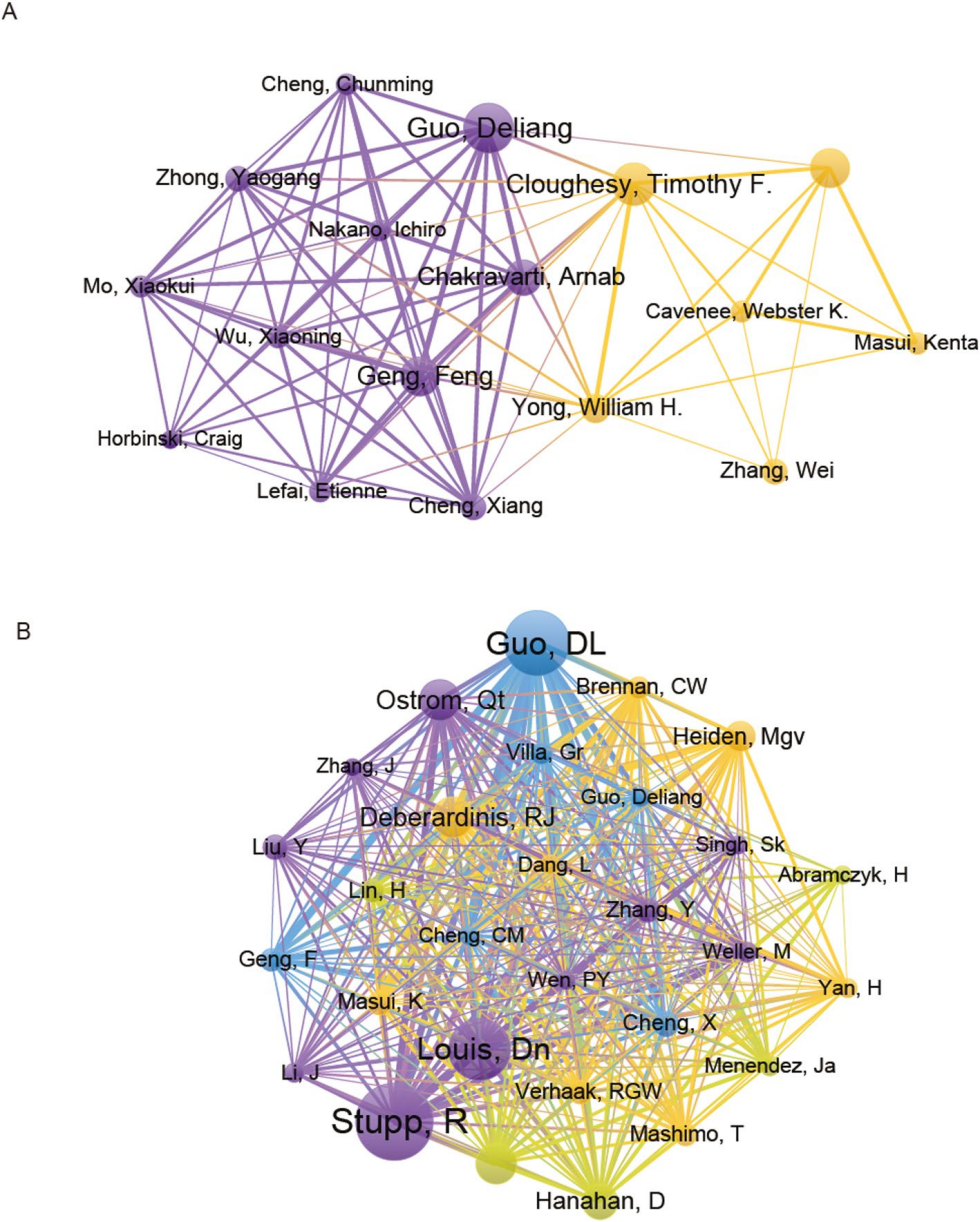
Author collaboration and co-citation networks. **A** Graph showing top authors and their co-authorship patterns. Prominent contributors include Deliang Guo, Zsolt Balogi, and Yongjun Kou. Denser clusters represent close-knit research groups. **B** Network of authors who are frequently co-cited, indicating shared thematic influence. Core nodes signify foundational figures in lipid metabolism and GBM

The co-authorship network highlights influential researchers, such as Deliang Guo, whose contributions to the sterol regulatory element-binding protein 1 (SREBP1) and Sterol O-Acyltransferase 1 (SOAT1) pathways have provided a foundation for further mechanistic studies. Other key figures include Yongjun Kou and Zsolt Balogi, who have examined lipid droplet dynamics and lipidomic alterations in GBM tissues. A collaborative analysis revealed that most productive author clusters are present within institutional networks, suggesting that dedicated research groups drive much of the innovation in this field (Fig. 7).

#### Co-authorship network analysis (Fig. 7A)

A co-authorship analysis revealed prominent collaborative relationships among researchers in the field of GBM lipid metabolism. Two major clusters emerged. The first was centered around Guo Deliang, Geng Feng, and Chakravarti Arnab, representing a highly interconnected group with frequent collaborations, particularly among researchers based in the United States and China. The second cluster was led by Cloughesy Timothy F., Cavenee Webster K., and Yong William H., and reflects a cohesive research team focusing on translational and clinical GBM studies. The strong intra-cluster links and several inter-cluster connections suggest a moderately collaborative global research environment, with cross-institutional and cross-national cooperation shaping the progress in this field.

#### Co-citation network analysis (Fig. 7B)

The co-citation analysis highlights the intellectual structure of GBM lipid metabolism studies. Guo D.L., Stupp R., Louis D.N., DeBerardinis R.J., Warburg O., and Ostrom Q.T. rank among the most highly co-cited and most strongly connected authors, indicating their influence in shaping our current understanding of lipid metabolism in GBM. Notably, Stupp R. is strongly associated with clinical advances in standard-of-care treatment, whereas DeBerardinis R.J. and Guo D.L. have contributed significantly to the elucidation of metabolic reprogramming in gliomas. The interconnections among these highly cited authors suggest a shared reliance on landmark studies and an emerging consensus on mechanistic and therapeutic targets within the field.

### Reference co-citation analysis

**Fig. 8:**
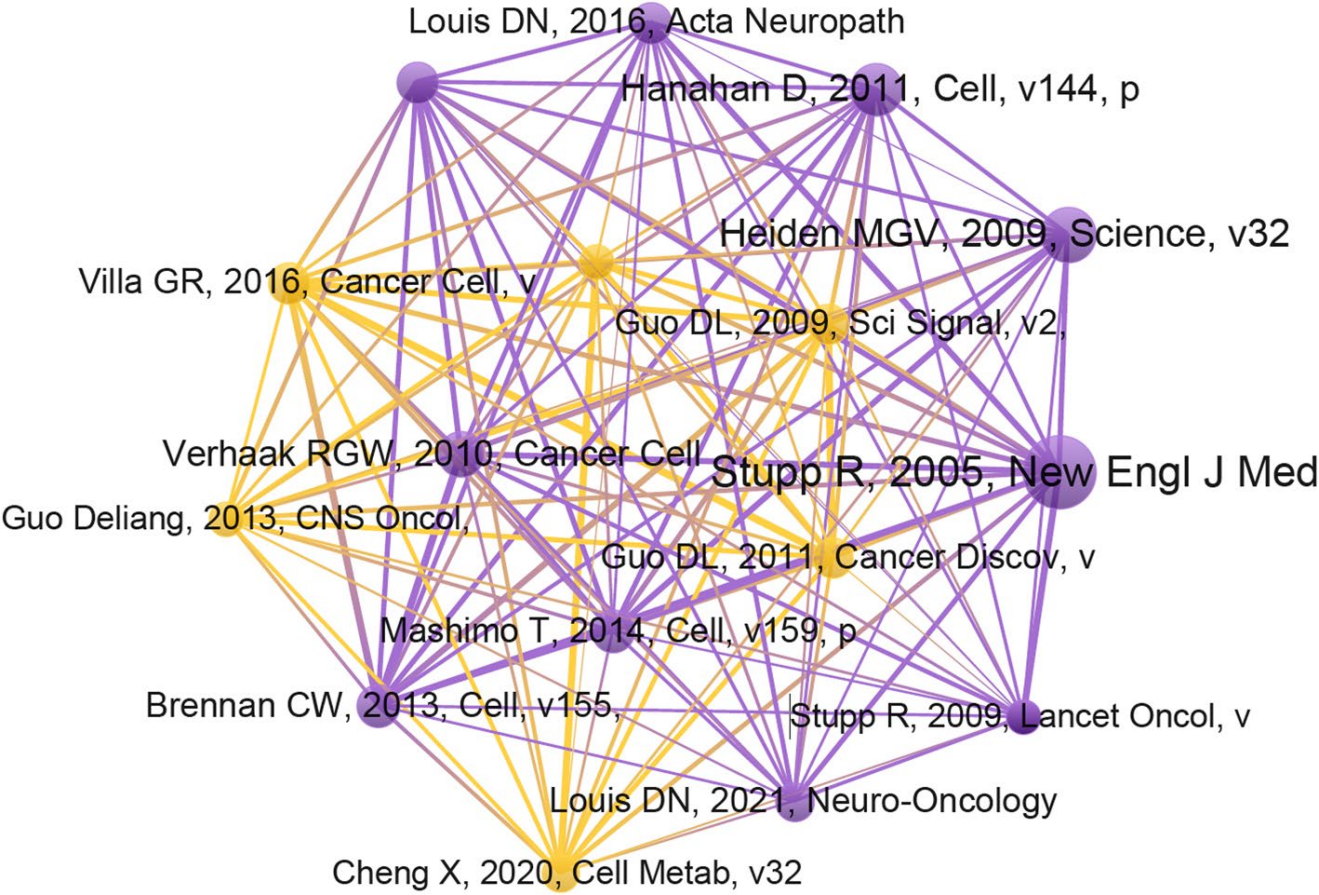
Co-citation network of references. Visualization of highly co-cited references, grouped into distinct clusters representing shared thematic relevance. Each node represents a frequently cited publication, and the size of the node reflects its citation frequency. Edges indicate co-citation relationships

Figure 8 presents a reference co-citation network, highlighting the foundational literature in the GBM lipid metabolism field. Notably, Stupp et al. (2005, New Engl J Med) is the most frequently co-cited article, reflecting its pivotal role in establishing the standard of care for GBM. Other highly co-cited studies include Louis et al. (2016, Acta Neuropathologica), which examines the molecular classification of gliomas, and Hanahan and Weinberg (2011, Cell), which examines hallmark cancer biology. Metabolism-focused articles, such as Heiden et al. (2009, Science) and Guo D.L. (2009, Sci Signal; 2011, Cancer Discovery), also feature prominently, thus highlighting the growing recognition of metabolic reprogramming in glioma pathogenesis. The dense clustering and cross-linking among references indicate a strong thematic coherence among molecular pathology, metabolism, and clinical therapeutics. Taken together, these citations represent the intellectual backbone of current GBM lipid metabolism research.

### Citation bursts and knowledge evolution

**Fig. 9:**
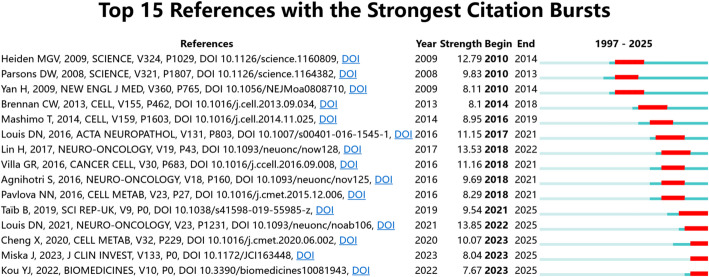
Citation burst detection for top references. Timeline of citation bursts showing emergent and declining research focuses

Figure 9 shows the top 15 references with the strongest citation bursts from 1997 to 2025, which reflect influential studies that garnered rapid attention during specific periods. Of these, Louis et al. (2021, Neuro-Oncology) had the highest burst strength (13.85), followed by Lin et al. (2017) and Heiden et al. (2009, Science), indicating their contributions to shaping the research trends in GBM lipid metabolism. Recent bursts (2022–2025) are dominated by publications, such as Cheng et al. (2020, Cell Metab), Miska et al. (2023, J Clin Invest), and Kou et al. (2022, Biomedicines), which demonstrate a growing emphasis on metabolic regulation and lipid biology in glioma research. Earlier high-impact studies, including Parsons et al. (2008, Science) and Yan et al. (2009, NEJM), provide a molecular foundation for glioma classification and mutation profiling.

### Keyword co-occurrence and thematic clustering

**Fig. 10:**
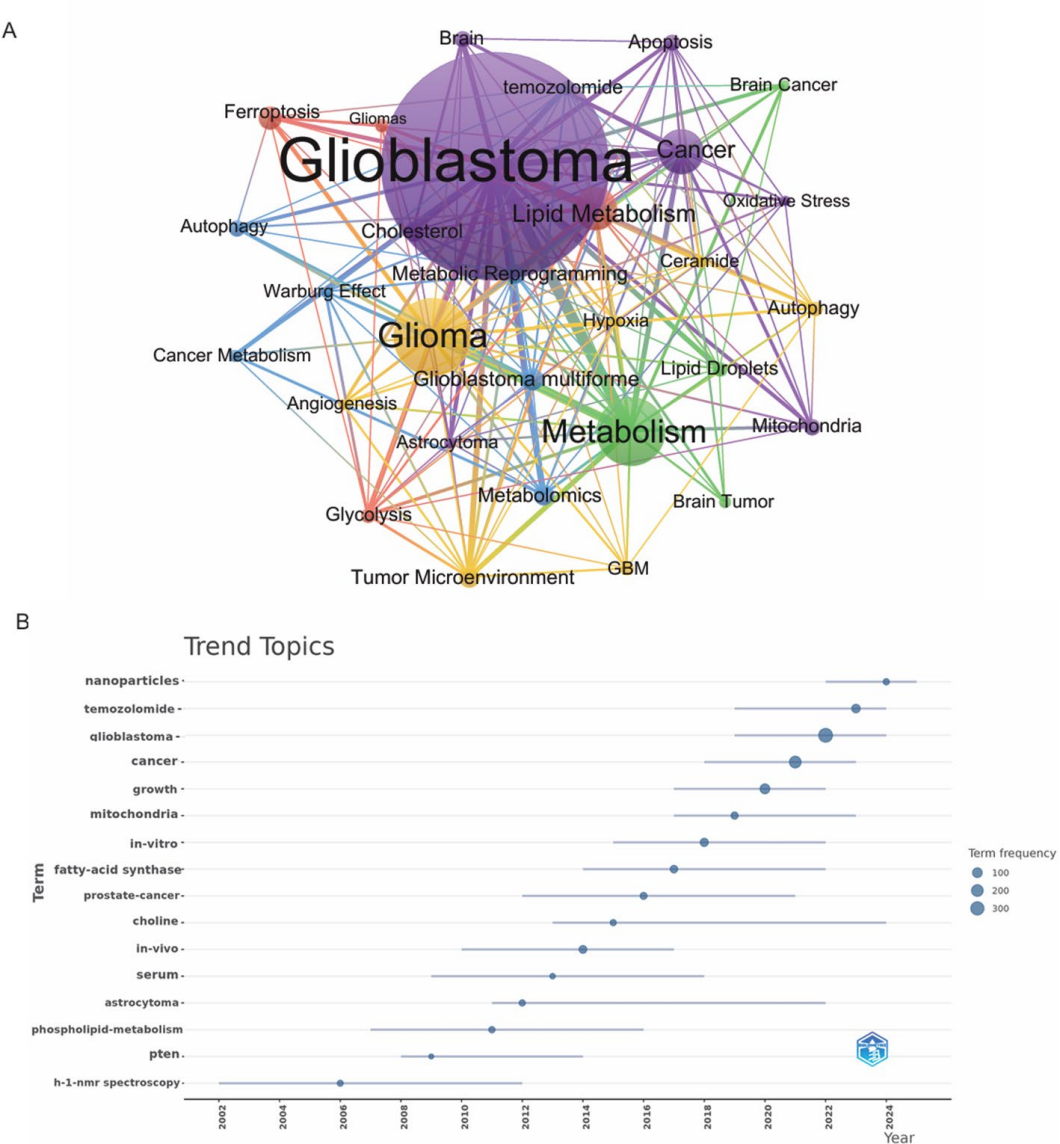
Author keyword co-occurrence network. Clustering map of frequently used author keywords. Major themes include “lipogenesis,” “lipid droplet,” “fatty acid oxidation,” and “immune regulation,” reflecting conceptual structure in the field

Keyword co-occurrence analysis revealed several interconnected thematic clusters in glioblastoma lipid metabolism research (Fig. 10A). Cluster 1 was anchored by terms, such as glioblastoma, temozolomide, and apoptosis, which reflect foundational interest in GBM pathogenesis and response to chemotherapy. Cluster 2 highlighted metabolic themes, including lipid droplets, metabolic reprogramming, ceramide, and mitochondria, which indicate a focus on altered lipid metabolism and organelle function in glioma. Cluster 3 featured tumor microenvironment, angiogenesis, Warburg effect, and hypoxia, representing studies on tumor ecology and metabolic adaptation. Cluster 4 included broader metabolic and disease-related terms, such as cancer metabolism, glycolysis, and oxidative stress. Temporal trend analysis (Fig. 10B) revealed the recent interest in nanoparticles, temozolomide, glioblastoma, and mitochondria, indicating evolving research aimed at nanotechnology-based delivery and mitochondrial targeting. In a GBM-restricted corpus, the trend-topic analysis was dominated by pathology/therapy and study-design terms (e.g., glioblastoma, temozolomide, nanoparticles, in vivo/in vitro), indicating that lipid metabolism research revolves around tumor biology and translational endpoints, rather than lipid biochemistry alone. After filtering generic disease/method terms, lipid-focused signals (e.g., choline, phospholipid metabolism, fatty-acid synthase, mitochondria) remained prominent, which provide a clearer view of lipid-metabolism–specific research trends. A keyword word cloud summarizing term frequency in the dataset is provided in Supplementary Figure S1.

## Discussion

GBM is one of the most aggressive and lethal forms of brain cancer, with a disproportionately high burden of cancer-related mortality [2, 31, 32]. Its resilience stems not only from its invasive nature, but also from its remarkable metabolic plasticity [33]. Our bibliometric and scientific analysis delineated the evolution and prospective trajectory of lipid metabolism studies in glioblastoma. In addition to highlighting the significance of lipid metabolism in GBM biology, the results indicated a shift of this research area from a specialized niche to a prominent focus within neuro-oncology. This progression is significant given the context of GBM as one of the most aggressive and lethal brain tumors, hallmarked by its invasiveness and notable metabolic plasticity [34].

Since approximately 2017, there has been a notable increase in publications, likely attributable to the growing recognition of metabolic vulnerabilities in GBM and the advent of enabling technologies, such as lipidomics, single-cell profiling, and advanced imaging techniques that have facilitated lipidomics research. Conceptually, the field has progressed beyond a glucose-centric perspective of cancer metabolism and has revealed lipids as central regulators [35, 36], further emphasizing lipid pathways as promising targets for therapeutic intervention [37–40].

Geographic and institutional patterns reveal a field that is characterized by global distribution alongside structural concentration. The United States retains a leadership position, particularly as a central node for international collaboration, whereas China demonstrates rapid growth in research output with networks that are comparatively more regionally concentrated. This asymmetry suggests that future advancements may be enhanced through more extensive cross-regional integration. The clustering of institutions around major academic centers further suggests that GBM lipid metabolism studies are driven by specialized groups with a sustained focus. Although this may accelerate progress, it also poses the risk of thematic silos if there is limited exchange between groups.

Journal and co-citation analyses revealed that lipid metabolism in GBM is inherently multidisciplinary and positioned at the intersection of oncology, metabolism, and molecular biology. The availability of clinically oriented and mechanistic journals has provided a reciprocal exchange flow between laboratory research and clinical application.

Keyword co-occurrence and clustering analysis (Fig. 10) indicated that the predominant themes continue to focus on general GBM biology, chemoresistance, and metabolic reprogramming. This indicates that lipid metabolism is primarily examined within the broader context of tumor adaptation, rather than as an independent field. Conversely, highly specialized topics, such as ferroptosis and immunometabolism, despite their biological potential, are less prominent in the bibliometric landscape, suggesting that they are emerging, rather than established themes.

The analysis of citation bursts (Fig. 9) corroborates this interpretation. Seminal publications on glioma classification and core metabolic reprogramming represent the most pronounced citation bursts, highlighting the enduring influence of foundational oncogenic and metabolic paradigms in guiding lipid-centric research. Recent citation bursts pertaining to metabolic regulation and mitochondrial function suggest a progressive refinement toward studies that are organelle-specific and pathway-focused. This progression indicates a shift in the field from broad metabolic descriptions to a more mechanistic precision.

The integration of overlay visualization and keyword clustering indicates a translational trajectory, with citation flows transitioning from molecular biology to clinical medicine. The emergence of terms such as nanoparticles and mitochondria in the recent literature suggests a shift from descriptive metabolic studies to therapeutic applications. This trend is consistent with the ongoing challenge of drug delivery across the blood-brain barrier (BBB). Strategies such as lipid nanoparticle carriers, focused ultrasound-mediated BBB disruption, and receptor-mediated transcytosis are being explored to improve central nervous system bioavailability [41]. Consequently, studies on lipid metabolism in GBM not only target metabolic pathways, but also explore delivery mechanisms for lipophilic or lipid-based interventions, which are otherwise constrained by pharmacokinetic challenges.

At the biological level, the literature reviewed here indicates that GBM relies on metabolic plasticity rather than a singular dominant lipid pathway. The SREBP1-driven de novo lipogenesis pathway is consistently highlighted, with downstream enzymes, such as FASN and ACC, playing important roles in membrane biogenesis and signal transduction [15]. Preclinical studies indicate that the pharmacological inhibition of SREBP1 or its downstream effectors may disrupt glioma stem cell maintenance and enhance sensitivity to therapy, thus demonstrating the translational significance of this pathway [11].

FAO, particularly under hypoxic conditions, plays a complementary role in supporting adenosine triphosphate (ATP) production and indirectly contributes to maintaining redox balance through pathways associated with nicotinamide adenine dinucleotide phosphate NADPH homeostasis [42]. Carnitine palmitoyltransferase 1 (CPT1) has been identified as an important regulatory node in this process [43]. Massalha et al. reported that Minerval induces cancer-selective toxicity in glioblastoma by uncoupling mitochondrial oxidative phosphorylation and exploiting tumor fatty-acid dependence [44]. In the literature, the roles of lipogenesis and FAO in GBM cells currently include anabolic and catabolic lipid pathways [11], suggesting that therapeutic strategies targeting various metabolic states may be more effective than focusing on a single pathway.

Another prominent theme is the accumulation of lipid droplets, which buffer excess fatty acids and enable metabolic flexibility during stress [45], whereas lipophagy facilitates rapid lipid mobilization [46, 47]. Regulators, such as adipose triglyceride lipase and perilipin-2 (PLIN2), are considered potential therapeutic targets [48, 49], thereby linking organelle biology to therapeutic sensitivity. For example, Lladó et al. found that pharmacologically remodeling glioma cell membrane lipid composition suppresses oncogenic signaling, which supports lipids as therapeutic targets [50].

In addition to well-established regulators, such as FASN and SREBP1, new therapeutic targets have been identified, such as stearoyl-CoA desaturase-1 (SCD1), lysophosphatidylcholine acyltransferase (LPCAT), and those involved in cardiolipin remodeling, which play important roles in modulating membrane fluidity, maintaining mitochondrial integrity, and regulating apoptosis. These relatively unexplored components present novel therapeutic opportunities [51].

Despite these findings, bibliometric analysis indicates that the translation of mechanistic discoveries into clinical applications remains insufficient. Although lipid-targeted strategies, such as inhibiting SREBP1-driven lipogenesis, carnitine palmitoyltransferase 1 (CPT1)-mediated FAO, or the induction of ferroptosis, are being evaluated in experimental settings, they are underrepresented in high-impact or frequently occurring keyword domains. This discrepancy indicates a translational gap between experimental potential and clinical implementation.

Ferroptosis, which is a non-apoptotic form of cell death characterized by iron-dependent lipid peroxidation, has gained prominence as a significant area of research, particularly concerning treatment-resistant, stem-like GBM cells [52]. Key regulators, including ACSL4, GPX4, and FSP1, are potential therapeutic targets [53]. Although ferroptosis is not yet a predominant term in bibliometric analyses, its mechanistic association with lipid peroxidation and therapy resistance highlights its importance as a significant emerging field [15]. Its relatively modest bibliometric presence likely indicates that it is an emerging, rather than a fully developed research theme.

The endocannabinoid system, which includes bioactive lipids such as anandamide and 2-arachidonoylglycerol, also plays a role in GBM lipid metabolism [54]. These lipid-derived signals attenuate glioma proliferation, immune cell polarization, and inflammatory responses through CB1 and CB2 receptors. Despite being relatively underexplored, this pathway presents significant translational potential, particularly in modulating tumor–immune interactions [55].

Immune-related aspects of lipid metabolism were also less prominently represented in the keyword trends, despite their strong biological rationale. This suggests that the study of immunometabolism in GBM remains at a relatively early stage of investigation, despite its considerable translational potential. Tumor-associated macrophages and microglia can accumulate lipids and adopt an immunosuppressive M2-like phenotype [24]. Lipid mediators, such as prostaglandins, oxidized phospholipids, and endocannabinoids, stabilize PD-L1, impair antigen presentation, and induce T-cell exhaustion. These mechanisms, in aggregate, may hinder the effectiveness of immunotherapy, thereby positioning immunometabolism as an important area of research [56].

### Limitations and future directions

Although this study has notable strengths, several limitations should be acknowledged. The exclusive reliance on the WoSCC may have resulted in the omission of pertinent studies contained in other databases. In addition, bibliometric indicators are susceptible to a citation lag, potentially leading to an underrepresentation of recent influential work. Keyword-based analyses are dependent on author-defined terms, which may introduce bias. Moreover, because bibliometric term extraction is frequency/co-occurrence driven, less standardized intervention phrases (e.g., “lipid therapy/replacement” or “membrane composition”) may not emerge as dominant topic labels, even when mechanistically relevant. Finally, our reliance on English-language publications and selected databases may introduce selection bias, as non-English studies, although few, were excluded and tend to receive fewer citations.

To address these challenges, future bibliometric analyses should incorporate datasets from PubMed, Scopus, and Embase. The integration of machine learning tools for semantic analysis can also assist in identifying latent thematic structures. Longitudinal tracking of co-citation networks may facilitate the prediction of emerging research hotspots. From a translational standpoint, further studies are needed to validate lipid-targeted therapies in preclinical and clinical settings. The use of patient-derived xenografts, 3D organoids, and immune-competent models will be important for translating laboratory findings into clinical applications. Furthermore, the identification of reliable biomarkers for patient stratification is an essential area of study.

### Implications for research and practice

Our review supports lipid metabolism as a pivotal element in the biology of GBM and its resistance to therapy. Incorporating lipidomic profiling into conventional molecular diagnostics may reveal metabolic dependencies that can be targeted, particularly in recurrent or treatment-resistant disease. Policymakers and funding agencies should consider prioritizing interdisciplinary initiatives that connect lipid biology, pharmacology, and neuro-oncology.

The field has progressed from observational studies to a comprehensive discipline that integrates molecular biology, systems-level omics, and translational science. The effect of lipid-targeted strategies will depend on sustained interdisciplinary collaboration and technological advancements. By addressing mechanistic gaps, enhancing drug delivery, and stratifying patients, we may unlock the therapeutic potential of targeting lipid metabolism in glioblastoma.

## Conclusion

This bibliometric study reveals the evolving research landscape of lipid metabolism in glioblastoma since the mid-1990s, with a marked increase after 2010 and a shift from descriptive metabolic profiling to mechanistic and early translational studies. Core research themes center on de novo lipogenesis, FAO, lipid droplet dynamics, immunometabolic regulation, and emerging hotspots, such as ferroptosis, endocannabinoid signaling, and lipid storage regulators. Despite strong preclinical evidence, clinical translation remains limited because of tumor heterogeneity, metabolic plasticity, and therapeutic delivery challenges. Future progress will likely depend on integrating lipidomics with spatial transcriptomics, immune profiling, and patient stratification, while underexplored areas, such as sex-specific lipid signatures and lipid–immune interactions, may reveal therapeutic opportunities. Overall, this analysis provides a structured overview of the current field and emerging directions in GBM lipid metabolism research, which will inform future studies and therapeutic advances in neuro-oncology.

## Supplementary Information

Below is the link to the electronic supplementary material.

Supplementary Material 1.

Supplementary Material 2.

## Data Availability

No datasets were generated or analysed during the current study.

## References

[1] Ostrom QT, Gittleman H, Farah P, Ondracek A, Chen Y, Wolinsky Y, Stroup NE, Kruchko C, Barnholtz-Sloan JS. CBTRUS statistical report: Primary brain and central nervous system tumors diagnosed in the United States in 2006–2010. Neuro Oncol. 2013;15(Suppl 2):ii1–56. 10.1093/neuonc/not151. 10.1093/neuonc/not151 PMC3798196 24137015

[2] Price M, Ballard C, Benedetti J, Neff C, Cioffi G, Waite KA, Kruchko C, Barnholtz-Sloan JS, Ostrom QT. CBTRUS Statistical Report: Primary Brain and Other Central Nervous System Tumors Diagnosed in the United States in 2017–2021. Neuro Oncol. 2024;26(vi1–vi85). 10.1093/neuonc/noae145. 10.1093/neuonc/noae145 PMC11456825 39371035

[3] Thomas DL. 2021 updates to the World Health Organization classification of adult-type and pediatric-type diffuse gliomas: a clinical practice review. Chin Clin Oncol. 2023;12. 10.21037/cco-22-120. 10.21037/cco-22-120 36922356

[4] Antonelli M, Poliani PL. Adult type diffuse gliomas in the new 2021 WHO Classification. Pathologica. 2022;114:397–409. 10.32074/1591-951X-823. 36534419 10.32074/1591-951X-823 PMC9763975

[5] Louis DN, Perry A, Wesseling P, Brat DJ, Cree IA, Figarella-Branger D, Hawkins C, Ng HK, Pfister SM, Reifenberger G, et al. The 2021 WHO Classification of Tumors of the Central Nervous System: a summary. Neuro Oncol. 2021;23:1231–51. 10.1093/neuonc/noab106. 34185076 10.1093/neuonc/noab106 PMC8328013

[6] Wesseling P, Capper D, Reifenberger G, Sarkar C, Hawkins C, Perry A, Kleinschmidt-DeMasters B, Komori T, Paulus W, Santosh V, et al. cIMPACT-NOW update 11: Proposal on adaptation of diagnostic criteria for IDH- and H3-wildtype diffuse high-grade gliomas and for posterior fossa ependymal tumors. Brain Pathol. 2026;36:e70035. 10.1111/bpa.70035. 40887057 10.1111/bpa.70035 PMC12695693

[7] Jezierzanski M, Nafalska N, Stopyra M, Furgol T, Miciak M, Kabut J, Gisterek-Grocholska I. Temozolomide (TMZ) in the Treatment of Glioblastoma Multiforme-A Literature Review and Clinical Outcomes. Curr Oncol. 2024;31:3994–4002. 10.3390/curroncol31070296. 39057168 10.3390/curroncol31070296 PMC11275351

[8] Darefsky AS, King JT Jr., Dubrow R. Adult glioblastoma multiforme survival in the temozolomide era: a population-based analysis of Surveillance, Epidemiology, and End Results registries. Cancer. 2012;118:2163–72. 10.1002/cncr.26494. 21882183 10.1002/cncr.26494 PMC3235223

[9] Schupper AJ, Hadjipanayis CG. Novel approaches to targeting gliomas at the leading/cutting edge. J Neurosurg. 2023;139:760–8. 10.3171/2023.1.JNS221798. 36840741 10.3171/2023.1.JNS221798 PMC11225597

[10] Badr CE, Silver DJ, Siebzehnrubl FA, Deleyrolle LP. Metabolic heterogeneity and adaptability in brain tumors. Cell Mol Life Sci. 2020;77:5101–19. 10.1007/s00018-020-03569-w. 32506168 10.1007/s00018-020-03569-w PMC8272080

[11] Lu L, Zhang Y, Yang Y, Jin M, Ma A, Wang X, Zhao Q, Zhang X, Zheng J, Zheng X. Lipid metabolism: the potential therapeutic targets in glioblastoma. Cell Death Discov. 2025;11. 10.1038/s41420-025-02390-3. 10.1038/s41420-025-02390-3 PMC11914282 40097417

[12] Fernandez-Garcia P, Malet-Engra G, Torres M, Hanson D, Rossello CA, Roman R, Llado V, Escriba PV. Evolving Diagnostic and Treatment Strategies for Pediatric CNS Tumors: The Impact of Lipid Metabolism. Biomedicines. 2023;11. 10.3390/biomedicines11051365. 10.3390/biomedicines11051365 PMC10216525 37239036

[13] Welte MA, Gould AP. Lipid droplet functions beyond energy storage. Biochim Biophys Acta Mol Cell Biol Lipids. 2017;1862:1260–72. 10.1016/j.bbalip.2017.07.006. 28735096 10.1016/j.bbalip.2017.07.006 PMC5595650

[14] Zeng Y, Zhao L, Zeng K, Zhan Z, Zhan Z, Li S, Zhan H, Chai P, Xie C, Ding S, et al. TRAF3 loss protects glioblastoma cells from lipid peroxidation and immune elimination via dysregulated lipid metabolism. J Clin Invest. 2025;135. 10.1172/JCI178550. 10.1172/JCI178550 PMC11957706 39932808

[15] Kou Y, Geng F, Guo D. Lipid Metabolism in Glioblastoma: From De Novo Synthesis to Storage. Biomedicines. 2022. 10.3390/biomedicines10081943. 36009491 10.3390/biomedicines10081943 PMC9405736

[16] Grube S, Dunisch P, Freitag D, Klausnitzer M, Sakr Y, Walter J, Kalff R, Ewald C. Overexpression of fatty acid synthase in human gliomas correlates with the WHO tumor grade and inhibition with Orlistat reduces cell viability and triggers apoptosis. J Neurooncol. 2014;118:277–87. 10.1007/s11060-014-1452-z. 24789255 10.1007/s11060-014-1452-z

[17] Geng F, Cheng X, Wu X, Yoo JY, Cheng C, Guo JY, Mo X, Ru P, Hurwitz B, Kim SH, et al. Inhibition of SOAT1 Suppresses Glioblastoma Growth via Blocking SREBP-1-Mediated Lipogenesis. Clin Cancer Res. 2016;22:5337–48. 10.1158/1078-0432.CCR-15-2973. 27281560 10.1158/1078-0432.CCR-15-2973 PMC5093025

[18] Kant S, Kesarwani P, Prabhu A, Graham SF, Buelow KL, Nakano I, Chinnaiyan P. Enhanced fatty acid oxidation provides glioblastoma cells metabolic plasticity to accommodate to its dynamic nutrient microenvironment. Cell Death Dis. 2020;11. 10.1038/s41419-020-2449-5. 10.1038/s41419-020-2449-5 PMC7170895 32312953

[19] Zhang J, Liu B, Xu C, Ji C, Yin A, Liu Y, Yao Y, Li B, Chen T, Shen L, et al. Cholesterol homeostasis confers glioma malignancy triggered by hnRNPA2B1-dependent regulation of SREBP2 and LDLR. Neuro Oncol. 2024;26:684–700. 10.1093/neuonc/noad233. 38070488 10.1093/neuonc/noad233 PMC10995519

[20] Miska J, Chandel NS. Targeting fatty acid metabolism in glioblastoma. J Clin Invest. 2023. 10.1172/JCI163448. 36594473 10.1172/JCI163448 PMC9797338

[21] Cheng X, Geng F, Pan M, Wu X, Zhong Y, Wang C, Tian Z, Cheng C, Zhang R, Puduvalli V, et al. Targeting DGAT1 Ameliorates Glioblastoma by Increasing Fat Catabolism and Oxidative Stress. Cell Metab. 2020;32(e228):229–42. 10.1016/j.cmet.2020.06.002. 32559414 10.1016/j.cmet.2020.06.002 PMC7415721

[22] Geng F, Zhong Y, Su H, Lefai E, Magaki S, Cloughesy TF, Yong WH, Chakravarti A, Guo D. SREBP-1 upregulates lipophagy to maintain cholesterol homeostasis in brain tumor cells. Cell Rep. 2023;42. 10.1016/j.celrep.2023.112790. 10.1016/j.celrep.2023.112790 PMC10528745 37436895

[23] Dai S, Yan Y, Xu Z, Zeng S, Qian L, Huo L, Li X, Sun L, Gong Z. SCD1 Confers Temozolomide Resistance to Human Glioma Cells via the Akt/GSK3beta/beta-Catenin Signaling Axis. Front Pharmacol. 2017;8. 10.3389/fphar.2017.00960. 10.3389/fphar.2017.00960 PMC5758607 29354058

[24] Governa V, de Oliveira KG, Bang-Rudenstam A, Offer S, Cerezo-Magana M, Li J, Beyer S, Johansson MC, Mansson AS, Edvardsson C, et al. Protumoral lipid droplet-loaded macrophages are enriched in human glioblastoma and can be therapeutically targeted. Sci Transl Med. 2024;16:eadk1168. 10.1126/scitranslmed.adk1168. 39475570 10.1126/scitranslmed.adk1168

[25] Pang L, Zhou F, Chen P. Lipid-Laden Macrophages Recycle Myelin to Feed Glioblastoma. Cancer Res. 2024;84:3712–4. 10.1158/0008-5472.CAN-24-3362. 39292810 10.1158/0008-5472.CAN-24-3362 PMC12165008

[26] Wu Y, Pu X, Wang X, Xu M. Reprogramming of lipid metabolism in the tumor microenvironment: a strategy for tumor immunotherapy. Lipids Health Dis. 2024;23. 10.1186/s12944-024-02024-0. 10.1186/s12944-024-02024-0 PMC10832245 38302980

[27] Manoj Kumar L, George RJ, P SA. Bibliometric Analysis for Medical Research. Indian J Psychol Med. 2023;45:277–82. 10.1177/02537176221103617. 37152388 10.1177/02537176221103617 PMC10159556

[28] Synnestvedt MB, Chen C, Holmes JH. CiteSpace II: visualization and knowledge discovery in bibliographic databases. AMIA Annu Symp Proc. 2005:724–728. PMC1560567 16779135

[29] van Eck NJ, Waltman L. Software survey: VOSviewer, a computer program for bibliometric mapping. Scientometrics. 2010;84:523–38. 10.1007/s11192-009-0146-3. 20585380 10.1007/s11192-009-0146-3 PMC2883932

[30] Arruda H, Silva ER, Lessa M, Proenca D Jr., Bartholo R. VOSviewer and Bibliometrix. J Med Libr Assoc. 2022;110:392–5. 10.5195/jmla.2022.1434. 36589296 10.5195/jmla.2022.1434 PMC9782747

[31] Brain GBD, Other CNSCC. Global, regional, and national burden of brain and other CNS cancer, 1990–2016: a systematic analysis for the Global Burden of Disease Study 2016. Lancet Neurol. 2019;18:376–93. 10.1016/S1474-4422(18)30468-X. 30797715 10.1016/S1474-4422(18)30468-X PMC6416167

[32] Grochans S, Cybulska AM, Siminska D, Korbecki J, Kojder K, Chlubek D, Baranowska-Bosiacka I. Epidemiology of Glioblastoma Multiforme-Literature Review. Cancers (Basel). 2022. 10.3390/cancers14102412. 35626018 10.3390/cancers14102412 PMC9139611

[33] Torrisi F, Alberghina C, D’Aprile S, Pavone AM, Longhitano L, Giallongo S, Tibullo D, Di Rosa M, Zappala A, Cammarata FP et al. The Hallmarks of Glioblastoma: Heterogeneity, Intercellular Crosstalk and Molecular Signature of Invasiveness and Progression. Biomedicines. 2022. 10.3390/biomedicines10040806 10.3390/biomedicines10040806 PMC9031586 35453557

[34] Darwish A, Pammer M, Gallyas F Jr., Vigh L, Balogi Z, Juhasz K. Emerging Lipid Targets in Glioblastoma. Cancers (Basel). 2024. 10.3390/cancers16020397. 38254886 10.3390/cancers16020397 PMC10814456

[35] Koundouros N, Poulogiannis G. Reprogramming of fatty acid metabolism in cancer. Br J Cancer. 2020;122:4–22. 10.1038/s41416-019-0650-z. 31819192 10.1038/s41416-019-0650-z PMC6964678

[36] Snaebjornsson MT, Janaki-Raman S, Schulze A. Greasing the Wheels of the Cancer Machine: The Role of Lipid Metabolism in Cancer. Cell Metab. 2020;31:62–76. 10.1016/j.cmet.2019.11.010. 31813823 10.1016/j.cmet.2019.11.010

[37] Cheng C, Geng F, Cheng X, Guo D. Lipid metabolism reprogramming and its potential targets in cancer. Cancer Commun (Lond). 2018;38. 10.1186/s40880-018-0301-4. 10.1186/s40880-018-0301-4 PMC5993136 29784041

[38] Gu D, Zhou F, You H, Gao J, Kang T, Dixit D, Wu Q, Yang K, Ci S, Shan D, et al. Sterol regulatory element-binding protein 2 maintains glioblastoma stem cells by keeping the balance between cholesterol biosynthesis and uptake. Neuro Oncol. 2023;25:1578–91. 10.1093/neuonc/noad060. 36934350 10.1093/neuonc/noad060 PMC10651206

[39] Jiang N, Xie B, Xiao W, Fan M, Xu S, Duan Y, Hamsafar Y, Evans AC, Huang J, Zhou W, et al. Fatty acid oxidation fuels glioblastoma radioresistance with CD47-mediated immune evasion. Nat Commun. 2022;13. 10.1038/s41467-022-29137-3. 10.1038/s41467-022-29137-3 PMC8938495 35314680

[40] Fernández-García P, Malet-Engra G, Torres M, Hanson D, Rosselló CA, Román R, Lladó V, Escribá PV. Evolving Diagnostic and Treatment Strategies for Pediatric CNS Tumors: The Impact of Lipid Metabolism. Biomedicines. 2023;11:1365. 37239036 10.3390/biomedicines11051365 PMC10216525

[41] Wang C, Xue Y, Markovic T, Li H, Wang S, Zhong Y, Du S, Zhang Y, Hou X, Yu Y, et al. Blood-brain-barrier-crossing lipid nanoparticles for mRNA delivery to the central nervous system. Nat Mater. 2025. 10.1038/s41563-024-02114-5. 39962245 10.1038/s41563-024-02114-5 PMC12514551

[42] Lin H, Patel S, Affleck VS, Wilson I, Turnbull DM, Joshi AR, Maxwell R, Stoll EA. Fatty acid oxidation is required for the respiration and proliferation of malignant glioma cells. Neuro Oncol. 2017;19:43–54. 10.1093/neuonc/now128. 27365097 10.1093/neuonc/now128 PMC5193020

[43] Kim SJ, Park SJ, Park J, Cho HJ, Shim JK, Seon J, Choi RJ, Yoon SJ, Moon JH, Kim EH, et al. Dual inhibition of CPT1A and G6PD suppresses glioblastoma tumorspheres. J Neurooncol. 2022;160:677–89. 10.1007/s11060-022-04189-z. 36396930 10.1007/s11060-022-04189-z

[44] Massalha W, Markovits M, Pichinuk E, Feinstein-Rotkopf Y, Tarshish M, Mishra K, Llado V, Weil M, Escriba PV, Kakhlon O. Minerval (2-hydroxyoleic acid) causes cancer cell selective toxicity by uncoupling oxidative phosphorylation and compromising bioenergetic compensation capacity. Biosci Rep. 2019. 10.1042/BSR20181661. 30602451 10.1042/BSR20181661 PMC6340956

[45] Wu X, Geng F, Cheng X, Guo Q, Zhong Y, Cloughesy TF, Yong WH, Chakravarti A, Guo D. Lipid Droplets Maintain Energy Homeostasis and Glioblastoma Growth via Autophagic Release of Stored Fatty Acids. *iScience* 2020, *23*, 101569. 10.1016/j.isci.2020.101569 10.1016/j.isci.2020.101569 PMC7549116 33083736

[46] Olzmann JA, Carvalho P. Dynamics and functions of lipid droplets. Nat Rev Mol Cell Biol. 2019;20:137–55. 10.1038/s41580-018-0085-z. 30523332 10.1038/s41580-018-0085-z PMC6746329

[47] Wang C, Haas MA, Yeo SK, Paul R, Yang F, Vallabhapurapu S, Qi X, Plas DR, Guan JL. Autophagy mediated lipid catabolism facilitates glioma progression to overcome bioenergetic crisis. Br J Cancer. 2021;124:1711–23. 10.1038/s41416-021-01294-0. 33723393 10.1038/s41416-021-01294-0 PMC8110959

[48] Zhang R, Meng J, Yang S, Liu W, Shi L, Zeng J, Chang J, Liang B, Liu N, Xing D. Recent Advances on the Role of ATGL in Cancer. Front Oncol. 2022;12. 10.3389/fonc.2022.944025. 10.3389/fonc.2022.944025 PMC9326118 35912266

[49] Li X, Kang K, Shen L, Shen L, Zhou Y. Integrative Analysis of the Predictive Value of Perilipin Family on Clinical Significance, Prognosis and Immunotherapy of Glioma. Biomedicines. 2023. 10.3390/biomedicines11041009. 37189627 10.3390/biomedicines11041009 PMC10136080

[50] Llado V, Lopez DJ, Ibarguren M, Alonso M, Soriano JB, Escriba PV, Busquets X. Regulation of the cancer cell membrane lipid composition by NaCHOleate: effects on cell signaling and therapeutical relevance in glioma. Biochim Biophys Acta. 2014;1838:1619–27. 10.1016/j.bbamem.2014.01.027. 24525074 10.1016/j.bbamem.2014.01.027

[51] Morais CM, Cardoso AM, Araujo ARD, Reis A, Domingues P, Domingues MRM, de Lima MCP, Jurado AS. Stearoyl CoA Desaturase-1 Silencing in Glioblastoma Cells: Phospholipid Remodeling and Cytotoxicity Enhanced upon Autophagy Inhibition. Int J Mol Sci. 2022. 10.3390/ijms232113014. 36361811 10.3390/ijms232113014 PMC9654881

[52] Yang WS, Stockwell BR, Ferroptosis. Death by Lipid Peroxidation. Trends Cell Biol. 2016;26:165–76. 10.1016/j.tcb.2015.10.014. 26653790 10.1016/j.tcb.2015.10.014 PMC4764384

[53] Sun H, Zhang J, Qi H, Jiang D, Hu C, Mao C, Liu W, Qi H, Zong J. Ioning out glioblastoma: ferroptosis mechanisms and therapeutic frontiers. Cell Death Discov. 2025;11. 10.1038/s41420-025-02711-6. 10.1038/s41420-025-02711-6 PMC12381129 40858533

[54] Holt S, Fowler CJ. Anandamide metabolism by fatty acid amide hydrolase in intact C6 glioma cells. Increased sensitivity to inhibition by ibuprofen and flurbiprofen upon reduction of extra- but not intracellular pH. Naunyn Schmiedebergs Arch Pharmacol. 2003;367:237–44. 10.1007/s00210-002-0686-z. 12644895 10.1007/s00210-002-0686-z

[55] Sanchez C, de Ceballos ML, Gomez del Pulgar T, Rueda D, Corbacho C, Velasco G, Galve-Roperh I, Huffman JW, Ramon y Cajal S, Guzman M. Inhibition of glioma growth in vivo by selective activation of the CB(2) cannabinoid receptor. Cancer Res. 2001;61:5784–9. 11479216

[56] Dumitru CA, Sandalcioglu IE, Karsak M. Cannabinoids in Glioblastoma Therapy: New Applications for Old Drugs. Front Mol Neurosci. 2018;11:159. 10.3389/fnmol.2018.00159. 29867351 10.3389/fnmol.2018.00159 PMC5964193

