# Supplementary material for: Status and trends in glioblastoma lipid metabolism research: a bibliometric analysis
Source: Discov Oncol. 2026 May 30;17:1106. doi: 10.1007/s12672-026-05298-1 (PMC13433873; doi:10.1007/s12672-026-05298-1)
Supplement: Supplementary file 1 [file 12672_2026_5298_MOESM2_ESM.docx]

**Supplementary Figure S1：The keyword word cloud of lipid metabolism on glioma.**
